# Unveiling metal-support interactions through interpretable machine learning

**DOI:** 10.1093/nsr/nwae455

**Published:** 2024-12-14

**Authors:** Junzhong Xie, Ding Ma

**Affiliations:** Beijing National Laboratory for Molecular Sciences, New Cornerstone Laboratory, College of Chemistry and Molecular Engineering, Peking University, China; Beijing National Laboratory for Molecular Sciences, New Cornerstone Laboratory, College of Chemistry and Molecular Engineering, Peking University, China

In 1978, Tauster *et al*. introduced the concept of strong metal-support interaction (SMSI) to describe the encapsulation of metal nanoparticles by suboxide supports, a phenomenon known to enhance catalyst resistance to sintering and degradation [1]. This discovery broadened the understanding of metal-support interactions (MSIs), drawing increasing attention to the field. Decades of research have revealed MSI as an effective approach for catalyst design, influencing the catalytic performance through charge transfer, chemical composition, perimeter sites, particle morphology and suboxide encapsulation [2]. In 2021, Wei-Xue Li's team proposed a volcano relationship between sintering tendency and MSI intensity [3]. However, fundamental questions persist around the nature of MSIs, due to complex interfacial bonding and structural variability [4].

In a recent article published in *Science*, ‘Nature of metal-support interaction for metal catalysts on oxide supports’ [5], Wei-Xue Li and colleagues made a breakthrough by integrating interpretable machine learning, domain knowledge, extensive experimental data and theoretical derivation to establish a general theory of MSIs for metal catalysts on oxide supports. They employed advanced symbolic regression techniques [6] to analyze historical experimental interfacial adhesion energies covering 25 metals and 27 oxides.

**Figure 1.**
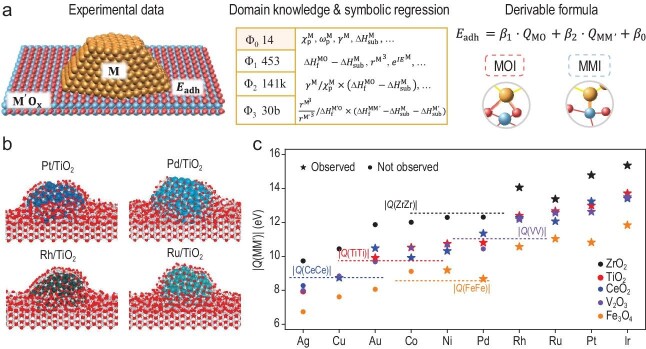
(a) Schemes of the identification of MSI formula through interpretable machine learning. (b) Molecular dynamic simulations of the encapsulation of the Pt, Pd, Rh and Ru clusters on TiO_2_(011) support. Red indicates O atoms and gray Ti atoms; other colors indicate supported metal atoms. (c) The $Q({\rm MM}^{\prime})$ for late transition metals on CeO_2_, TiO_2_, ZrO_2_, Fe_3_O_4_ and V_2_O_3_ with corresponding ${\rm M}^{\prime}$ affinity of ${\rm M}^{\prime}$ in oxide, $Q({\rm M}^{\prime}{\rm M}^{\prime} )$ (horizontal dashed lines). Reproduced with permission from ref. [5].

This approach led to the discovery of an accurate and derivable formula for MSIs, which decouples metal-oxygen interaction (MOI) and metal-metal interaction (MMI) contributions from basic properties of the materials (Fig. 1a), and allows for the prediction of MSIs for 675 metal-oxide systems. Except for the contributions from the MOI term, the MMI term is a surprisingly novel and important quantity whose role was largely unrecognized before. Further large-scale molecular dynamic simulations based on neural network potentials revealed that MMI also dictates the kinetic rates of the encapsulation process in SMSI and the proportion of metal-metal bonds at the encapsulation interface (Fig. 1b). Based on these findings, a principle of strong MMI for the occurrence of SMSI was proposed, which not only explains nearly all observed encapsulation phenomena for 10 metals and 16 oxides, but also predicts new systems yet to be discovered (Fig. 1c), such as Ir on more oxides and late transition metals on manganese oxide and chromium oxide. This finding highlights the role of MMI in determining support effects, offering a new avenue for understanding and engineering the support catalysts.

The proposed new theory exhibits broad applicability. It applies not only to oxide-supported metal nanoparticle catalysts but also to metal single-atom catalysts and metal-supported oxide catalysts. Additionally, because the theory is built based on basic properties, it has the potential to be extrapolated to more catalytic systems, such as nitride and carbide supports. The developed MSI theory therefore offers a constructive guideline to engineer interfaces between metals and supports for the design of efficient catalysts. Importantly, the exemplified integration of interpretable AI algorithms with domain knowledge to build mathematical formulas and develop scientific principles offers a novel perspective for scientific discovery in the era of ‘AI for Science’.
